# Validation of an Eco-Friendly Automated Method for the Determination of Glucose and Fructose in Wines

**DOI:** 10.3390/molecules28145585

**Published:** 2023-07-22

**Authors:** Irene Dini, Dario Tuccillo, Daniele Coppola, Margherita-Gabriella De Biasi, Elena Morelli, Andrea Mancusi

**Affiliations:** 1 Department of Pharmacy, University of Naples Federico II, Via Domenico Montesano 49, 80131 Napoli, Italy; margherita.debiasi@unina.it (M.-G.D.B.); elena.morelli@unina.it (E.M.); 2 Lcm Laboratorio Chimico Merceologico, Corso Meridionale, 80143 Napoli, Italy; dario.tuccillo@gmail.com (D.T.); d.coppola@si-impresa.na.camcom.it (D.C.); 3 Department of Food Microbiology, Istituto Zooprofilattico Sperimentale del Mezzogiorno, Via Salute 2, 80055 Portici, Italy

**Keywords:** automated colorimetric test, fermentable sugars dosage, metrological approach, food analysis

## Abstract

Fermentable sugar dosage helps oenologists to establish a harvest’s moment and control the fermentation process of the musts. The official analyses recommended for their determination are long, laborious, and must be carried out by specialized personnel. On the contrary, instrumental analysis automation limits human errors, increases precision, and reduces the time and cost of the analyses. In the food production sector, to use methods other than those recommended by supranational bodies in official reports, it is necessary to validate the analytical processes to establish the conformity of the results between the new methods and the reference ones. This work validated an automated enzymatic apparatus to determine the sum of glucose and fructose levels in wine samples. The validation was carried out on wine samples (dry red wine, dry white wine, moderately sweet wine, and sweet wine) containing different sugar concentrations by comparing data obtained using the OIV-MA-AS311-02 method performed by a specialized operator (reference method) and the same method performed by an automated apparatus. The difference between the results’ means obtained with the two procedures was significant. Nevertheless, the automated procedure was considered suitable for the intended use since the differences between the averages were lower than the measurement uncertainty at the same concentration, and the repeatability results were better for the automated procedure than the reference method.

## 1. Introduction

The total sugars (fructose and glucose) range in grapes is between 150 and 300 g/L [1].

The sugar levels in the grape are of interest to the wine industry to predict wine quality. Their concentrations in grapes impact the optimum harvest time to ripen grapes, regulate the fermentation process, and define the wine’s product class. European Legislation, according to the total sugar content, classifies wines into semidry (>4–12 g/L), dry (≤4 g/L), sweet (>45 g/L), and semi-sweet (>12–45 g/L) (Commission Regulation, 2009) [2]. Glucose and fructose are mono-saccharides with the same empirical formula (C_6_H_12_O_6_) and different structures. They are contained in equal amounts in grapes. The sugar levels and composition change during grape maturing and can be affected by a moderate water deficit, low temperatures (below 30 °C), and UV-B radiation [3,4,5,6]. During wine fermentation, glucose and fructose are transformed by yeasts (e.g., Saccharomyces cerevisiae) in carbon dioxide, ethanol, glycerol, etc. [7]. They impact the wine alcoholic degree between 10 and 15% [8], the wine’s perception of mouthfeel (e.g., astringency, viscosity, and density), and texture [9]. The sugars can decrease the astringency perception (interacting with polyphenols), retain the wine aroma compounds, and improve the colloidal wine stability [10]. Must with high sugar content has a sluggish and complex alcoholic fermentation, with even fermentation stops [11]. Glucose ferments more than fructose since *Saccharomyces cerevisiae* yeasts prefer to ferment glucose [12]. The residual fructose level in fermented musts can impact the risk of microbial spoilage of the finished wine and gives an undesirable sweetness perception to the dry wines, as the fructose is approximately twice sweeter than glucose [13]. Several procedures have been reported in the literature to determine the fermentable sugar level analysis. Unfortunately, references methods are generally titrimetric procedures (such as the Lane–Eynon method included as a reference method in AOAC methods [14]), which must be developed by expert personnel, requires lengthy analysis times, meticulous control of reaction conditions, and consumption of reagents harmful to the environment [15,16,17,18]. The spectroscopic evaluation of fermentable sugar levels in wines (recommended by the International Organisation of Vine and Wine) is more accessible to perform than titrimetric methods. However, in any case, it is strongly affected by the measurement uncertainty linked to the working capacity of the operating personnel. This method is based on the phosphorylation of D-glucose and D-Fructose with ATP, catalyzed by hexokinase, and the subsequent oxidation of the glucose-6-phosphate and fructose-6-phosphate with nicotinamide adenine dinucleotide phosphate (NADP) in the presence of glucose-6-phosphate dehydrogenase to give NADPH and D-glucono-1,5-lactone 6-phosphate according to the reaction:

D-glucose 6-phosphate + NADP^+^ → D-glucono-1,5-lactone 6-phosphate + NADPH ^+^H^+^

The amount of NADPH corresponds to the amount of glucose and fructose present in the sample and is determined spectrophotometrically by measuring its absorbance at λ 340 nm, before and after the addition of NADP^+^ and the glucose-6 enzyme-phosphate dehydrogenase.

The present work aims to validate the employment of an automated apparatus to perform the OIV-MA-AS311-02 analysis to streamline the food product laboratories’ work and avoid human errors [19,20]. The automated apparatus used for the sugar dosage is an open analyzer that carries out analyses with low operating costs (minimizes user intervention), environmental impact (requires small volumes of reagents and samples and low water consumption), and minimal maintenance. The lab work is sped up by allowing for continuous loading of samples and reagents. The possibility of using dedicated reagents further reduces the possibility of operator error. The automated analyzer allows for the samples’ incubation at a controlled temperature. The highly selective enzymatic reaction allows for the detection of glucose and fructose in spectrum fields without interference. Using an automated apparatus reduces the need for specialized operators and the analysis risks due to human error. As reported in UNI CEI EN ISO/IEC 17025: 2017, a robust validation procedure is essential when changes are made to official methods [21,22] to confirm that it is still suitable for the intended purpose. The main objective of validation is to estimate the measurement uncertainty, a parameter considered an “indicator” of the correspondence to the purpose and the traceability of the results. The parameters to be validated are the measuring range (the range of values of the measurand within which precision and accuracy take on values acceptable), robustness (defined as the ability possessed by a method of not being affected, significantly, in terms of final results due to deliberate variations introduced in its implementation phases; intermediate repeatability tests (control charts) give information on the robustness of the method and can report the need to set up an actual study for robustness estimation), specificity (the ability to quantitatively measure properties chemical properties of a measurand in the presence of potential interferents), and sensitivity (the ability to discriminate between slight differences in the concentration or quantity or characteristics of the measuring) [23]. The validation procedures are lengthy and laborious and can be performed by highly specialized operators. Therefore, the scientific community should take charge of validating innovative analytical methods to allow for an adaptation of the reference analysis procedures to the new technologies offered by the market.

## 2. Results and Discussion

The concentration of fermentable sugars in grapes is susceptible to environmental stress. In wines, they determine organoleptic characteristics that influence the product class. The International Organisation of Vine and Wine (OIV) recommends the enzymatic spectrophotometric OIV-MA-AS311-02 method [18] (performed by an operator) to determine the sum of glucose and fructose in wines.

In this work, an automated apparatus was validated to perform the OIV-MA-AS311-02 method. 

The validation plan verified the compliance of the automated apparatus’s results with those obtained by the specialized operators (reference method). 

According to ISO/IEC (2005) [24], it was validated in terms of linearity, LOD (limits of detection) and LOQ (limit of quantification), accuracy, and precision. 

**Figure 1:**
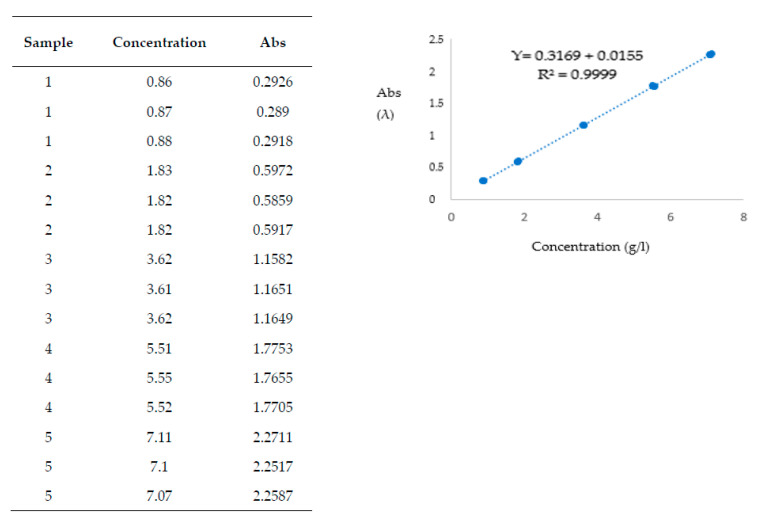
Calibration curve.

**Figure 2:**
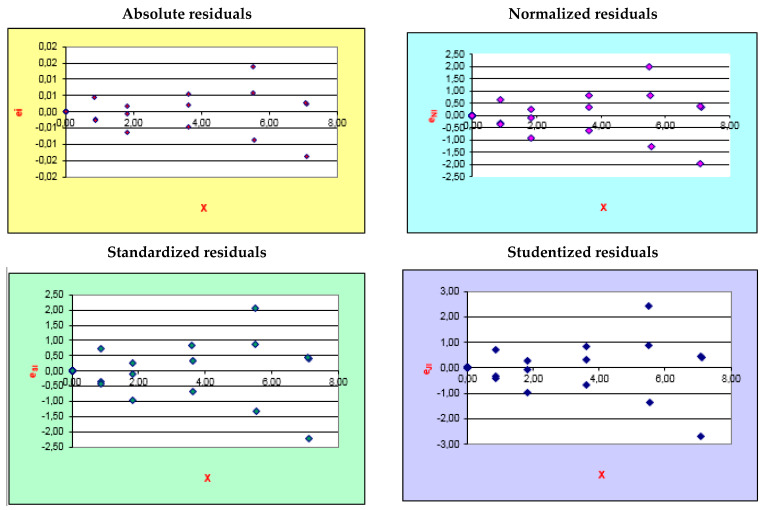
Residual distribution evaluated via ANOVA test. ei = absolute residuals; eNi = normalized residuals); eSi = studentized residuals; eji = standardized residuals.

The coefficient of determination (R^2^ = 0.9992) and the normal residual distribution in the ANOVA test confirmed the linearity of the calibration curve [25] (Figure 1 and Figure 2). The range in which the concentrations and spectrophotometric measurements were proportional was obtained by evaluating the calibration curve’s determination coefficient (R^2^) and the residues’ distributions.

Generally, R^2^ can vary between 0 and 1. When it is 0, the model does not explain the data; when it is 1, it describes the data perfectly [25].

The ANOVA test proved that there was normal residual distribution (Figure 2). The hypothesis was valid since the variance between the observed and expected values at each level was less than 10%. The coefficient of determination R^2^ = 0.9992 and the normal residual distribution in the ANOVA test confirmed the linearity of the calibration curves [26] (Figure 1 and Figure 2). The residual values in a regression analysis represent the prediction error portion of the regression model. The residuals, also called deviations, represent the differences between the values observed in the dataset and the estimated values calculated with the regression equation. The adequacy of the linear model is verified by analyzing the characteristics of the residuals studentized that correspond to transformations of the residuals (given by the difference between the values of the dependent variable detected and those estimated using the regression model). Under standard conditions, these Student residuals are distributed as Student’s t with n−h−1 degrees of freedom, where h corresponds to the number of parameters present in the model. When the points are arranged randomly and do not show “regularities”, the linear model is appropriate. The assumption of normality is verified through the analysis of the standardized residuals, which must be distributed, as increases, according to a standardized normal. In our case, more than 98% of the standardized residuals oscillated between −2 and +2. From the analysis of the studentized residuals, it was possible to exclude the presence of outliers (points due to erroneous measurements), which could have influenced the slope of the line (high values are considered those greater than 3 or less than −3). From the absolute residual plot, it was possible to exclude violations of the homoskedasticity assumption (collection of random variables with the same finite variance) [26].

The method sensitivity was evaluated via LOD, LOQ, and measuring range.

The LOD is the lowest analyte level in a sample that can be detected (not necessarily dosed) exactness. The LOQ is the lowest analyte level that can be dosed in a sample. The analytical test’s range is the interval between the upper and lower analyte levels for which the test is linear, precise, and accurate. It was determined by considering the linear range and LOQ.

LOD = 0.05 g/L LOQ = 0.08 g/L Decision limit: Ydc = 0.01 Xdc = 0.02

The fructose and glucose levels are variable in wines. High-quality wines generally have low levels (0.71 ± 0.73 and 0.32 ± 0.44 g/L, respectively) [12]. Sweet and sparkling wines have high dosages (can be less than 1.23 and 4.97 g/L) [27]. Therefore, to ensure that the method’s confidence limits encompassed the variability range of fermentable sugar content in all wines, the automated apparatus method precision was appraised by employing four wine samples to cover the entire measurement range: dry red wine (glucose and fructose content < 5 g/L), dry white wine (glucose and fructose content < 5 g/L), moderately sweet wine (glucose and fructose content in the range of 5–12 g/L), and sweet wine (glucose and fructose content > 12 g/L). Precision estimates the concordance among the results of subsequent measurements of the same quantity. It can be appraised by evaluating the measures’ repeatability and reproducibility. Repeatability (intra-assay precision) tests the agreement between the results of measurements performed under the same conditions in a short time (e.g., different days, operators, apparatus). Reproducibility examines the precision over time (inter-assay precision) by evaluating the results obtained on different days and by different laboratories (inter-laboratories precision).

Intra-assay precision was measured by performing ten analyses’ replicates of each reference wine on the same day; inter-assay precision was measured by replicating ten analyses of each reference wine on diverse days of a week (Table 1).

The difference between the values indicating a 95% coverage (greater than 0.05) was considered significant (Table 2).

Finally, was evaluated the method selectivity (to respond uniquely to the required analyte) by estimating the uncertainty associated with potential interferences. The values’ dispersion that could be attributed to the concentration measurement is defined as uncertainties. Measurement uncertainty is a quantitative value assigned to the measure. A change in this value causes a change in the associated uncertainty.

The method bias was estimated by evaluating “random” and “non-random” uncertainties in the measurement process. Uncertainties were calculated with the holistic method (method containing statistical data) [23]. 

The validity of the calibration curve for the state of the reagents used was evaluated by inserting the certified multisugar standard (4.0 g/L) in each work session and verifying that the method range acceptability was between 3.78 and 4.22 g/L. When this condition did not occur, the measure of the “blank” was repeated, or “blank and the calibration curve” were performed. The calibration status over time was appraised by controlling the process stability based on the points arranged above and below the central line (zero difference between the nominal concentration of the controls and the concentrations read by the instrument) and within the limits of acceptability (3.78–4.22 g/L) in the control charts (Levey–Jennings graphs provided by automated apparatus, which stores the analyzed samples’ batches in sheets in which data and statistical calculations are reported). Some causes considered responsible for random uncertainties are reported in Table 3.

The “non-random” uncertainties were estimated by comparing the results obtained by our laboratory with those of other laboratories participating in the Ring tests coordinated by the “Italian Union of Wine” (frequency 3/year). The circuits have about 300 participants (wineries, laboratories, public bodies, and research institutes). They are proficiency testing-type schemes, helpful in evaluating the performance of the testing laboratory and ensuring better control and quality of the results. The results’ standard deviations, obtained by the procedure performed by the automated apparatus, is lower than that reported by the test performed by an operator, proving that the two methods were superimposable.

## 3. Materials and Methods

### 3.1. Parameters and Measurement Ranges

The operating instruction was applied to the test method, OIV-MA-AS311-02 [18], determination of glucose and fructose in wines in a range 0–160.0 g/L (maximum concentration for a moderately sweet wine, sweet wine found by the historian of laboratory).

### 3.2. Samples

Four commercial Italian wine samples were analyzed. Four wine samples were chosen to cover the entire measurement range: dry red wine (glucose and fructose content < 5 g/L), dry white wine (glucose and fructose content < 5 g/L), moderately sweet wine (glucose content and fructose in a range of 5–12 g/L) and sweet wine (glucose and fructose content > 12 g/L). All the wine samples were protected from light exposure until analysis.

### 3.3. Chemicals 

Glucose and fructose analytical standards were provided by Sigma-Aldrich (Inc. 115 St. Louis, MO, USA).

A glucose/fructose commercial calibrator (contained standards at five concentration levels: 0.90, 1.80, 3.60, 5.40, and 7.20 g/L) was bought from Biosystems (Barcelona, Spain).

Kit for D-glucose/D-fructose analysis was obtained from Biosystems (Sinatech SY2404, Milan, Italy). 

R1 (2 × 40 mL) contained TRIS buffer (200 nM, pH 7.0), ATP (4 mM), NADP (3 mM), and sodium azide (<0.1%).

R2 (2 × 18 mL) contained HK (>0.5 UI/l), G6PDH (>1.8 UI/l), and PGI (>8 UI/l).

Milli-Q water was made with a Millipore purification system (Billerica, MA, USA). 

Instrument washing solution (deproteinizing) was supplied by Biosystem (Biosystem B013416; Milan, Italy). It was used diluted 1/200 with distilled water.

System liquid was purchased from Biosystems (Biosystems BO11524; Milan, Italy). It was used diluted (6 mL of liquid of the concentrated solution to the container filled with distilled water) after 12 h from preparation to reduce air bubbles.

### 3.4. Enzymatic Method 

The sum of glucose and fructose levels was performed according to the OIV-MAAS311-02 [18], both manually and into the automated sequential analyzer Y15 Biosystems (Sinatech, Grottazzolina (FM), Italy).

#### 3.4.1. Operating Modes

Each sample was processed 10 times with the spectrophotometer (λ 340 nm; Shimadzu SP UV 1800, Kyoto, Japan) and 10 times with Y15 Sinatech Biosystems (Biosystem B013416; Milan, Italy). Cloudy samples were filtered (paper filters for wine Polsinelli MFC0018.25, Broccostella (FR) Italy). Samples rich in CO_2_ were degassed using a water-jet vacuum pump (Gea; Parma, Italy).

The calibration curve was obtained using a glucose/fructose commercial calibrator (containing standards at five concentration levels: 0.90, 1.80, 3.60, 5.40, and 7.20 g/L). Three reads of the absorbance at λ 340 were carried out for each point.

##### Reference Method OIV-MA-AS311-02 (Manual Method)

The analyses were performed using Kit for D-glucose/D-fructose analysis obtained from Biosystems (Milan, Italy). The reagents were ready to use and stable until the expiration date when stored at 2–8 °C.

The kits whose blank (read at λ340) exceeded 0.500 OD were not used, as suggested by the manufacturer. Two cuvettes were prepared to perform the analyses, one for the sample and one for the blank.

In the blank cuvette, Reagent 1 (750 μL) and distilled water (9 mL) were mixed and incubated at 37 °C for 1 min, and then the absorbance was read at λ 340 (A1).

In the sample cuvette, Reagent 1 (750 μL) and sample (9 mL) were mixed, incubated at 37 °C for 10 min, and then the absorbance was read at λ 340 (A1).

Successively, Reagent 2 (750 μL) was added in both cuvettes, mixed, and cubed at 37 °C for 10 min, and the absorbance was read at λ 340 (A2).

$$[\mathrm{glucose}+\mathrm{fructose}]=\frac{\left(A2-0.84\times A1\right)sample-\left(A2-0.84\times A1\right)blank}{\left(A2-0.84A1\right)standard-\left(A2-0.84\times A1\right)blank}\times C\;g/L$$

0.84 correction factor used to correct the absorbance after the addition of Reagent 2, C concentration value D−GlucoseD−Fructose shown in the Multical wine table.

##### Automated Method OIV-MA-AS311-02

The analyses were performed using Kit for D-glucose/D-fructose analysis obtained from Biosystems (Milan, Italy). The reagents, blank, and sample were inserted into the automatic equipment. The parameters for setting the automated apparatus are shown in Table 4.

Y15 analyzer automatically dilutes the samples that exceed the method’s linearity limits (up to 8.0 g/L), as reported in Table 5.

### 3.5. Method Validation

The use of the automated apparatus to dosage fermentable sugars was validated in terms of linearity, LOD (limits of detection) and LOQ (limit of quantification), accuracy, and precision according to the ISO/IEC (2005) [23].

The test sensitivity was apprised by studying the calibration curve equation.

The calibration curve was obtained using triplicate spectrophotometric lecture of each standard in Biosystem’s commercial calibrator supplies (Biosystem B013416; Milan, Italy).

The sample concentrations tested proportional to those of the analyte were deduced from R^2^ ≅1.

The detection (LOD) and quantification limits (LOQ) were calculated as follows:

$$\mathrm{LOD}=3\;\times \;\frac{Sa\;(Standard\;deviation\;intercept)}{b\;\left(slope\;of\;the\;calibration\;curve\right)}\;$$

$$\mathrm{LOQ}=10\;\times \;\frac{Sa\;(Standard\;deviation\;intercept)}{b\;\left(slope\;of\;the\;calibration\;curve\right)}$$

The automated apparatus method’s precision and accuracy were appraised by employing four wine samples to cover the entire measurement range: dry red wine (glucose and fructose content < 5 g/L), dry white wine (glucose and fructose content < 5 g/L), moderately sweet wine (glucose and fructose content in the range of 5–12 g/L), and sweet wine (glucose and fructose content > 12 g/L).

The method precision was obtained by measuring intraday repeatability (ten replicates of each reference wine on the same day) and interday reproducibility (ten replicates of each reference wine on diverse days during a week).

Repeatability (r) = 0.056 × [concentration of glucose or fructose] in g/L

Reproducibility (R) = 0.12+0.076 [concentration of glucose or fructose] in g/L

The range of acceptability = C ± (0.056 × C)
C = nominal concentration of the standard
(0.056 × C) = method repeatability.

The method’s repeatability was verified by measuring inter-laboratory and intra-laboratory uncertainties (holistic method).

Intra-laboratory uncertainty = I = k × SR = 2 × SR

SR = reproducibility’s standard deviation coverage factor (k = 2)

Interlaboratory precision and trueness were evaluated by participating in Ring tests coordinated by the Italian Union of Wines and double tests (frequency 3/year).

### 3.6. Statistical Analysis

Statistical analyses (ANOVA and T-test) were performed with StatSoft software version 7.0 (StatSoft, Hamburg, Germany). Values of *p* < 0.05 were considered significant.

One-way analysis of variance (ANOVA) was employed to compare the two groups. T-test was employed to determine differences among results obtained by manual and automated procedures.

## 4. Conclusions

The work validated an automated apparatus to dosage fermentable wine sugars via the OIV-MA-AS311-02 reference method [18]. The validation process evaluated (in wines with different sugar levels) the OIV-MA-AS311-02 method performance when performed by a specialized operator (as required by the official method) and by automated apparatus. Statistical analysis performed using a T-test showed significant differences between the two tests. Nevertheless, the automated apparatus was considered suitable for the intended use since the differences between the averages of the two tests were lower than the measurement uncertainty calculated, and the repeatability was better for the automated method than the manual one. The results obtained from the validation process allow for using the apparatus tested for the legal analyses of the fermentable sugars levels in wines instead of the reference methods. These new technologies, often, as in this case, allow for considerable cost savings (reducing the use of specialized personnel and allowing for the performance of a more significant number of analyses per day than traditional methods), decrease the possibility of error due to the operator, and, above all, allow for more environmentally friendly analyses, optimizing the volumes of solvents required. The scientific community should take charge of validating innovative analytical methods to allow for an adaptation of the reference analysis procedures to the new technologies offered by the market.

## Figures and Tables

**Table 1:** Repeatability and reproducibility parameters.

DRY RED WINE		
	Automated method Abs	Manual method Abs
	2.64	2.447
	2.655	2.48
	2.621	2.44
	2.624	2.435
	2.643	2.402
	2.652	2.355
	2.622	2.434
	2.579	2.373
	2.603	2.398
	2.587	2.405
	Automated method	Manual method
Average (x_m_)	2.623	2.417
Standard deviation	0.026	0.037
Repeatability		0.105
Degrees of freedom	18	
Method repeatability		0.135
r/r(M)		0.779
Method reproducibility		0.304
Uncertainty		0.429
T experimental	13.534	
P	0.000	
T critical	2.100922	
**DRY WHITE WINE**		
	Automated method Abs	Manual method Abs
	1.678	1.679
	1.658	1.66
	1.651	1.661
	1.652	1.639
	1.65	1.725
	1.637	1.623
	1.64	1.726
	1.636	1.745
	1.663	1.756
	1.64	1.738
	Automated method	Manual method
Average (x_m_)	1.651	1.695
Standard deviation	0.013	0.048
Repeatability		0.136
Degrees of freedom	18	
Method repeatability		0.095
r/r(M)		1.435
Method reproducibility		0.249
Uncertainty		0.352
T experimental	2.684	
P	0.000	
T critical	2.10092204	
**MODERATELY SWEET WINE**		
	Automated method Abs	Manual method Abs
	7.08	7.187
	7.051	7.256
	6.986	7.235
	6.95	7.137
	6.919	7.29
	6.955	7.101
	6.959	7.317
	7.053	7.243
	6.995	7.378
	7.012	7.346
	Automated method	Manual method
Average (x_m_)	6.996	7.249
Standard deviation	0.052	0.089
Repeatability		0.251
Degrees of freedom	18	
Method repeatability		0.406
r/r(M)		0.619
Method reproducibility		0.671
Uncertainty		0.949
T experimental	7.360	
P	0.000	
T critical	2.100922	
**SWEET WINE**		
	Automated method Abs	Manual method Abs
	31.767	32.541
	31.09	33.125
	30.262	32.823
	31.793	32.979
	31.325	32.751
	31.583	31.077
	31.124	34.02
	31.197	31.683
	31.582	31.463
	31.912	32.483
	Automated method	Manual method
Average (x_m_)	31.364	32.495
Standard deviation (S)	0.485	0.874
Repeatability		2.471
Degrees of freedom	18	
Method repeatability		1.820
r/r(M)		1.358
Method reproducibility		2.590
Uncertainty		3.662
T experimental	3.396	
P	0.003	
T critical	2.100922	

**Table 2:** Summary table of the validation of method precision.

	Sample	Concentration g/L	*T*-Test Result	Uncertainty	
				Automated Method	Manual Method
1	red wine	<5 g/L	Significant difference	0.052	0.074
2	white wine	<5 g/L	Significant difference	0.026	0.096
3	moderately sweet wine	5–12 g/L	Significant difference	0.104	0.356
4	sweet wine	>5 g/L	Significant difference	0.97	1.748

**Table 3:** Some criteria for establishing the lack of process control.

Event	Possible Causes	Decision To Be Taken
A point is out of control limits.	The inexperience of the operator’s ex-pired check or incorrect conservation of the same.	Repeat the analysis; if the point is within the limit of control continues, otherwise stop, locate, and resolve the cause.
Seven consecutive points are above or below the central line	Defective kit control or incorrect con-servation of the same	If the eighth point falls on the side opposite to the line central continue, otherwise stop, locate, and resolve the cause.
Seven consecutive points are in ascending order (derive positive)	Obsolescence of reagents, progressive evaporation of solvent from the standard solution	If the eighth point changes, the order continues; otherwise, stop, locate, and resolve the cause.
Seven consecutive points are in descending order (derives negative)	Solution obsolescence, standards or reagents	If the eighth point changes, the order continues; otherwise, stop, locate, and resolve the cause.

**Table 4:** Automated method operative condition.

METHOD	
Sample volume (µL)	3
Reactive 1	250
Reactive2	50
Wash	1.2
Abs (nm)	340
Reading 1	72 s
Reading 2	600 s
Reactive 2	96 s
Temperature (°C)	37
CALIBRATION	
Calibration	Multiple calibrations
Calibrate replicates	3
Blank replicates	3
OPTIONS	
Blank limit (Abs; nm)	0.300
Linearity limit (g/L)	8

**Table 5:** Dilution factor applied by the apparatus for wines that exceed the method’s limits.

Concentration Range (g/L)	Dilution Factor
0–8.00	0
8.00–16.00	2
16.00–32.00	4
32.00–88.00	11
88.00–160.00	20

## Data Availability

The data presented in this study are available on request from the corresponding author.

## Sample Availability

Samples of the compounds are available from the authors.
